# Advanced genetic techniques in fungal pathogen research

**DOI:** 10.1128/msphere.00643-23

**Published:** 2024-03-12

**Authors:** Robbi L. Ross, Felipe H. Santiago-Tirado

**Affiliations:** 1 Department of Biological Sciences, University of Notre Dame, Notre Dame, Indiana, USA; 2 Eck Institute for Global Health, University of Notre Dame, Notre Dame, Indiana, USA; 3 Warren Center for Drug Discovery, University of Notre Dame, Notre Dame, Indiana, USA; University of Guelph, Guelph, Canada

**Keywords:** fungal pathogen, fungal genetics, *Cryptococcus*, *Candida*, *Aspergillus*

## Abstract

Although fungi have been important model organisms for solving genetic, molecular, and ecological problems, recently, they are also becoming an important source of infectious disease. Despite their high medical burden, fungal pathogens are understudied, and relative to other pathogenic microbes, less is known about how their gene functions contribute to disease. This is due, in part, to a lack of powerful genetic tools to study these organisms. In turn, this has resulted in inappropriate treatments and diagnostics and poor disease management. There are a variety of reasons genetic studies were challenging in pathogenic fungi, but in recent years, most of them have been overcome or advances have been made to circumvent these barriers. In this minireview, we highlight how recent advances in genetic studies in fungal pathogens have resulted in the discovery of important biology and potential new antifungals and have created the tools to comprehensively study these important pathogens.

## INTRODUCTION

Almost 5 years ago, we wrote about how the adaptation of classical yeast genetic techniques to the fungal pathogen *Cryptococcus neoformans* had transformed the field and ushered an era of accelerated discovery (1). Now, we write to highlight how recent cutting-edge genetic techniques, still mostly based on model yeast *Saccharomyces cerevisiae*, keep impacting the cryptococcal field and have transformed the studies of other fungal pathogens, including emerging ones such as *Candida auris*. At the time, we predicted that the establishment of advanced genetic techniques in *C. neoformans* would influence other fungal researchers and promote their implementation, or variations thereof, in their respective fields. With the ever-increasing incidence of fungal infections, the lack of effective treatments, and the emergence of fungal superbugs such as *C. auris*, these “genetic innovations” in the medical mycology field are necessary if we are to tackle, and win, the looming threat of fungal infections.

## WHY SHOULD WE STUDY FUNGI?

Fungi are everywhere, and we are exposed to them on a daily basis. Recent estimates suggest there are as many as 3.8 million species worldwide (2), although only around 300 species have been identified as pathogenic to humans, and fewer yet can cause disease in healthy individuals (3). Because of their ubiquitous nature and frequent exposure, this otherwise small number of fungal pathogens affect over 1 billion people, causing 300 million serious infections resulting in about 3.8 million deaths yearly (4). Most of these fungi are opportunistic pathogens and can only cause disease in individuals with compromised immune systems; hence, these infections have mostly been associated with HIV/AIDS patients. However, the incidence of these infections in the non-HIV population has been steadily increasing in the last decade due to (i) advances in medical treatments and procedures and (ii) climate change and industrialization.

Medical advancements such as solid-organ transplantation, radiation and chemotherapy, and treatments for autoimmune and inflammatory diseases, have led to an increase in the number of medically immunosuppressed individuals each year, all of which are highly susceptible to fungal infections (5). Nevertheless, fungal pathogens can also cause disease in individuals with various chronic illnesses or other infections, such as asthma, COPD, diabetes, liver disease, tuberculosis, influenza, and COVID-19 (6). Furthermore, accumulating evidence suggests that climate change and urbanization not only increase the endemic area of many pathogens but can directly impact and increase the pathogenic potential of fungal species (7, 8). Histoplasmosis, blastomycosis, and coccidioidomycosis, traditionally restricted to specific areas in the United States (*Histoplasma* along the Mississippi river basin, *Coccidioides* in the Southwest, and *Blastomyces* in the Midwest and the Central South), are now found throughout the continental United States (9). Additionally, there is evidence suggesting that *C. auris*, first described in a patient in 2009, became a human pathogen in part due to global warming (10). Because most fungi are adapted to ambient temperature, they cannot survive at physiological temperature. However, with global warming, fungi are adapting to higher temperatures, allowing them to survive and thrive in warm-blooded hosts. Recent outbreaks of emerging fungal pathogens, such as *Exserohilum rostratum* (11), *Fusarium solani* (12), and *C. auris* (13), highlight the inherent pathogenic potential of the fungal kingdom that can result in unexpected, growing fungal diseases worldwide. Moreover, many common pathogenic species are now becoming resistant to the limited number of treatments available (14). Prompted by all of these factors that have contributed to an increase in fungal infections, the World Health Organization (WHO) released its first-ever fungal priority pathogens list (FPPL) to systematically prioritize and raise awareness of these fungal pathogens (15)(Table 1). Fungal species in the critical group of the FPPL include *C. neoformans*, *Candida albicans*, *C. auris*, and *Aspergillus fumigatus*, which collectively affect millions of people, and each has mortality rates as high as >70% in vulnerable patient populations (Table 1). Thus, it is essential to study these, and related, fungal pathogens since they are a growing and prominent threat to public health (16).

**TABLE 1:** Information about the fungi in the critical group of the FPPL and the genetic tools mentioned in this review^*a*^

Species	Health burden	Selected genetic tools available	Sources
*C. neoformans*	120,000 deaths annually in the HIV+ population alone. Accounts for 19% of all AIDS-related deaths.	Optimized CRISPR/Cas9 editing system. Single-gene deletion collection. AK-luciferase high-throughput screening assay. Agrobacterium-mediated insertional mutagenesis.	(17–21)
*C. auris*	Mortality rates ranging from 30% to 72%.	Single-cell transcriptomic platform (mDrop-Seq). Optimized CRISPR/Cas9 editing system. Agrobacterium-mediated insertional mutagenesis.	(22–25)
*A. fumigatus*	2,100,000 estimated annual incidence, with mortality rates ranging from 30% to 90%.	AK-luciferase high-throughput screening assay. Optimized CRISPR/Cas9 editing system. Universal plasmids to facilitate gene deletion and tagging. High-throughput gene replacement.	(4, 26, 27)
*C. albicans*	995,000 deaths annually with a mortality rate of up to 71%.	CRISPR/Cas9 “gene drive.” Single-gene deletion collection. Non-editing CRISPR systems (CRISPRi and CRISPRa). Gene replacement and conditional expression libraries (GRACE). AK-luciferase high-throughput screening assay. High-throughput image analysis protocol. Single-cell transcriptomic platforms (mDrop-Seq, YeastDrop-Seq). Agrobacterium-mediated insertional mutagenesis.	(4, 24, 28–34)

^*a*^ *C. glabrata* is also discussed in this review but is not part of the FPPL’s critical group; hence, it is not included in the table.

## WHY ARE FUNGAL DISEASES SO CHALLENGING TO TREAT?

The lack of prophylactic and scarcity of therapeutic options for fungal infections has resulted in increased infection rates and prevented long-term control efforts. Human fungal pathogens are frequently overlooked, as such, research into their biology and pathogenesis has lagged behind that of bacteria and viruses (35). This is most clearly observed when comparing available treatment options. There are over two dozen classes of drugs used to treat bacterial infections, whereas there are only three classes currently available in the clinic for treating invasive fungal infections (36, 37). And yet, these few options are even more limited because they are expensive, are toxic, or are associated with both acquired and intrinsic resistance (38). Furthermore, some of these drugs are unavailable globally, especially in low-resource countries where the burden of fungal infections is the highest (39). In addition to a lack of antifungals, deficits in standardized guidelines for diagnosis and widespread clinical awareness have contributed to the rise and persistence of fungal infections (6, 40). Fungal infections are severely underdiagnosed, mainly due to the non-specific symptoms of severe disseminated and invasive infections and the propensity of physicians to look for bacterial or viral causes of disease (41). The delay in accurate diagnosis causes increased variability in patient prognosis, with some patients succumbing to infection before a proper diagnosis is made. Thus, due to the multitude of barriers regarding effective diagnosis and antifungal treatment, it is no surprise that these infections persist and are on the rise.

More research on pathogenic fungi needs to be conducted to address this global health concern and gain a better understanding of fungal biology to aid in the development of novel therapeutics and diagnostic tools (35). Most of the current antimicrobial treatments and robust diagnostic methods have come from genetic studies, thus making this research critical for our understanding of fungal biology (42, 43). Genetic research allows scientists to uncover gene function, identify novel drug targets, and discover novel virulence determinants in all microbial pathogens, including fungi. Currently, there are significant gaps in the knowledge of fungal biology and genetics, mostly a result of the traditional lack, or insufficient development, of genetic methods and tools and the limited range of high-throughput and molecular technologies (16). These difficulties in the study of fungal genetics have resulted in delays in identifying novel antifungal targets and developing new diagnostics, negatively impacting the global burden of fungal infections (16). However, in the last decade, this has changed, with exciting discoveries coming from the adaptation or development of cutting-edge genetic and molecular tools on a variety of important fungal pathogens, including the critical ones on the WHO’s FPPL.

## WHY ARE GENETIC STUDIES DIFFICULT IN FUNGI?

Intrinsic features of different fungal species make them difficult to study. Many pathogenic fungi have thick cell walls which serve as a barrier, making it difficult to study certain cellular processes and attain the genomic material for in-depth studies such as single-cell RNA sequencing (scRNA-seq) (44, 45). In addition, the fungal pathogen *C. neoformans* has an extensive capsule and a propensity for undergoing non-homologous recombination, both of which halted early genetic studies of this species (46). For added genetic complexity, fungal species can exist in different ploidy states. Some species exist as stable haploid, diploid, or polyploid cells, while other species can change ploidy depending on the present environment (47). For example, *C. neoformans* strains are typically found in the haploid state, whereas the most common human fungal pathogen, *C. albicans*, is typically found as diploid cells, making it difficult for genetic tools that rely on ploidy to be used between different fungal species (47). Despite their stable ploidy, polyploid strains of both species have been isolated in different environments, including from human patients both before and after antifungal treatment (48–50). Similarly, while most *Aspergillus* species are haploid, diploid clinical isolates of species such as *Aspergillus nidulans* have been isolated (51). The fact that changes in ploidy are frequently seen in both environmental and clinical isolates showcases their genetic complexity and ability to rapidly change the ploidy state under different conditions, highlighting why performing genetic research on these pathogens was more challenging than one would expect. However, for most of the main fungal pathogens, stable haploid laboratory strains have been established, and for others, like *C. albicans*, efforts are underway to generate or induce a haploid strain (52). In fact, the discovery that *C. albicans*, long thought to be an obligate diploid, was able to generate mating competent haploid strains was a gamechanger (53). Also, genetic techniques such as high-throughput scRNA-seq take advantage of the large size of mammalian cells to capture and lyse a single cell in oil droplets (54). The same was difficult to perform in pathogenic fungi because of their small size, tough cell walls, and smaller numbers of transcripts per cell when compared with multicellular organisms (55). Lastly, few medically important fungi have natural plasmids, which resulted in a lack of cloning vectors and ways to introduce DNA into most fungal pathogens. Thus, many factors make genetic research in pathogenic fungi difficult, making it more important that novel methods and tools continue to be developed to ease the study of these important microorganisms.

## RECENT EXAMPLES OF REVOLUTIONARY FUNGAL GENETICS RESEARCH

### What has been done previously?

Many methods, developed during the early 2000s using *S. cerevisiae*, revolutionized the field of yeast genetics, including the generation of the first complete gene knockout collection of any organism (56). This was accomplished because of budding yeast’s highly efficient homology-directed repair, along with its stable haploid state and ease of growth and manipulation. However, those very same attributes that made this budding yeast the genetics workhorse of the century are all missing in most medically important fungal pathogens. The first genomic resources in fungal pathogens were developed in *C. neoformans* and *C. albicans* and included partial collections of single-gene deletions (17, 33). This was shortly followed by a collection of 128 *A*. *nidulans* kinases in 2013 (57) and other partial collections in *C. albicans* such as the GRACE library (31). The contributions of these resources were monumental to the field of fungal genetics as it provided not only an incredible tool for researchers but also a plethora of useful data about fungal biology and disease. Improvements in genomic sequencing facilitate efforts to systematically delete genes and create knockout collections for more fungal pathogens or to create other resources such as fusing all genes to fluorescent proteins or affinity tags for biochemical purification. Lastly, the application of CRISPR for the genetic manipulation of microbial pathogens made its way to the field of fungal pathogens in 2015, with initial reports of feasibility in *Cryptococcus*, *Candida*, and *Aspergillus* (18, 23, 58). Overall, many tools have been developed in the past decade to progress fungal genetics research; however, with the persistence and rise in the incidence of fungal infections, innovative and novel approaches must continue to be created, or adapted, to break down barriers preventing the development of novel antifungals and diagnostic methods.

### Recent work showcasing cutting-edge fungal genetics

#### Development of CRISPR/Cas9 toolkits for fungal pathogens

Implementation of CRISPR/Cas9 approaches in fungal pathogens was slow and mostly limited to proof-of-concept studies. This has rapidly changed in the last few years, with the optimization of CRISPR/Cas9 constructs for rapid and efficient gene editing but also for non-editing applications such as CRISPR interference (CRISPRi) and CRISPR activation (CRISPRa). This is of great importance for the study of species that lack genetic collections but also for the study of essential genes, which are not present in those collections. Here, we summarize recent developments in CRISPR/Cas9 technologies in the major human fungal pathogens: *C. neoformans*, *C. albicans*, *C. auris*, and *Candida glabrata* (recently renamed *Nakaseomyces glabrata*).

*C. neoformans* is a globally distributed environmental yeast and a leading cause of death in the HIV+ population, with ~120,000 deaths in 2020 in this population alone (19). Despite several reports of CRISPR/Cas9 in this fungus, all were time-consuming, requiring the assembly of several large DNA fragments, including the repair template which needed long (~1 kb flanking arms) homology sequences. In 2022, Huang et al. optimized and developed a CRISPR/Cas9 system that allows for rapid genomic editing in *C. neoformans*, requiring only 50 bases of homology sequences, greatly facilitating the applicability of this technology (20). To achieve this, they developed a cryptococcal-optimized Cas9 expression cassette (CnoCas9) that showed greater Cas9 expression than all other Cas9 versions available at the time for genetic manipulation in *C. neoformans*. By targeting the *ADE2* and *URA5* loci, they showed that their CnoCas9 expression system allows for precise and efficient deletion via short homology-derived homologous recombination. To compare transient and stable CnoCas9 expression, they integrated this expression system into a “Safe Haven” locus of the fungus’s genome and showed that, while not affecting the virulence potential, this integration drastically increases transformation and homologous recombination efficiency. Furthermore, they showcased the usefulness of their CnoCas9 system by designing and implementing a combined localization and purification tag for *Cryptococcus*. Using this, they were able to tag various genes with a codon-optimized mNeonGreen fluorescent protein and showed a cellular localization consistent with the known function of the genes. Thus, this genetic tool is useful not only for reliable studies regarding genetic manipulation but also for studying localization and cryptococcal cell biology. Overall, their method allows the mediation of genetic manipulation using as short as 50 base pair regions of homology and only transient expression of sgRNA and Cas9 components. Although they only used the serotype A *C. neoformans* strain in their study, the authors have high expectations that their expression system can have broader implications since the previously used cryptococcal Cas9 expression system from *Caenorhabditis elegans* had activity in other *Cryptococcus* species. This is a significant development in cryptococcal genetics that will pave the way to even more complex and powerful assays such as unbiased CRISPR-Cas9 screens.

*C. albicans* is the most common and important medically relevant fungal pathogen, with over >1.5 million invasive infections annually, resulting in 995,000 deaths (4). Given the medical and academic relevance of this fungus, *C. albicans* was also one of the first fungal pathogens where CRISPR editing was shown to be feasible; however, due to alternative codon usage and the diploid status of this species, it was very inefficient. Still, it provided a platform for optimization, which resulted in the development of a “gene drive” by Shapiro et al. to rapidly generate homozygous single mutant diploids (28). This technique can even be used to generate homozygous double-gene deletion mutants by mating two strains, each containing a gene drive for specific target genes. This was, in turn, possible because of the earlier discovery of mating competent haploid strains of *C. albicans*, as mentioned above (53). This allows for the rapid generation of large libraries of mutants consisting of single- and double-gene deletions, useful if you want to study particular gene families, such as adhesin genes (59). Excitingly, using these optimized systems in *C. albicans* as the foundation, non-editing CRISPR systems were also generated. The first CRISPRi system fused a transcriptional repressor to a dead Cas9 enzyme, which results in the targeting of the repressor to specific locations in the genome (29). This was instrumental for the field given that other methods to study the function of essential genes, such as RNAi-based knockdown, do not work in this species. Most recently, again based on *S. cerevisiae*, the Shapiro group developed a CRISPRa system that requires just one large DNA molecule, can be applied to diverse strains, including clinical strains, and can be used for *in vivo* studies (30). They constructed an integrating plasmid containing all the necessary factors for CRISPRa: a dead Cas9, a tripartite gene activation complex, and an easy-to-clone Golden Gate site for the sgRNA sequence. One of the advances of this system is that it is stably integrated into the genome, requiring no constant selection, which allows for *in vivo* studies that otherwise would not be possible. The authors showed that integration of this plasmid does not affect the fungal fitness and can be successfully used over a variety of *C. albican*s strains, including clinical strains, that sometimes are refractory to transformation. They demonstrated that optimal overexpression can be achieved by targeting either the transcription start site or the transcription start codon, but that varies by gene. Lastly, through RNA-seq over time, they showed that there were no off-target effects even on similar genes, such as when targeting a member of a large gene family that are all similar to each other. Just as with the CRISPRi, the development of this CRISPRa system paves the way for overexpression-suppression screens or for other non-medical applications of *Candida* species, such as the production of economically important metabolites.

*C. auris* is an emerging fungal pathogen that has received much attention recently given that it is thought to be the first human pathogen to emerge due to global warming and has caused several, and ongoing, outbreaks all over the developed world (10, 13). Given its importance, it was not surprising that in the original paper describing CRISPR/Cas9 editing in *C. albicans*, there was a proof-of-concept experiment demonstrating that it was applicable to *C. auris* (23). However, the application of CRISPR/Cas9 tools in this fungus was hampered further due to low transformation efficiency, low rates of targeted integration of constructs, and significant efficacy variability in genetic manipulation between the different *C. auris* clades. These obstacles were addressed in 2021 by the O’Meara group when they developed not only transformation and forward genetic approaches in this fungus (discussed below) but also a broadly applicable CRISPR-Cas9 system for all *C. auris* clades (22). Similar to the optimized *Cryptococcus* system discussed above, the optimized system for *C. auris* consisted of the use of *C. auris* promoters, codon optimization of the Cas9 gene, and modification of the sgRNA scaffold by adding the *C. auris* tRNA-ALA sequence and the HDV ribozyme sequence that resulted in increased expression of functional gRNA. Using a fluorescent reporter to measure efficiency, they showed efficient targeted editing in all major clades that was as high as 80% in clade II. Moreover, they engineered a plasmid with the dominant selectable marker G418, allowing the use of this tool in any *C. auris* strain, irrespective if nutritional markers were present or not.

Lastly, *C. glabrata* is the second most common *Candida* species found in the clinical setting, and its study is emphasized by the rising antimicrobial resistance exhibited by this fungus. Since this fungus is more closely related to *S. cerevisiae* than other *Candida* species, CRISPR editing was first shown here in 2016, with subsequent optimizations and additions introduced in the following years (60–62). However, no CRISPRi system was available until recently, when it was developed by Billerbeck and colleagues as part of a broad *C. glabrata* genetic toolkit (63). The authors used several of the DNA molecules already generated for the toolkit to assemble a dead Cas9 fused to a repressor domain and a gRNA on a separate plasmid. They tested their system targeting two genes with known phenotypes and found that in many of the guides tested (four out of nine gRNA tested), they could confirm the expected phenotypes. Although it requires two vectors rather than one, this development represents a strong foundation for future optimizations of the system. With all of the recent CRISPR advancements accelerating mycology research, we expect that other model fungal pathogens will follow suit, as in this new report by PK Chang on a CRISPR-Cas9 with broad applicability in the *Aspergillus* genera (26).

#### Development of high-throughput screening assays in molds and non-model yeasts

One of the many reasons *S. cerevisiae* is still the model of choice for the screening of large compound libraries is the ease of high-throughput analyses in this yeast, be it genetic, functional, or morphological. Given the structural and morphological differences between yeasts and molds, genetics and high-throughput assays in the latter have lagged considerably relative to yeasts. *A. fumigatus* is the most common cause of invasive mold infections, with mortality rates ranging from 60% to 90% (27). Mold-active antifungals have limited efficacy due to significant toxicity, drug-drug interactions, and emerging resistance. To address this, Beattie and Krysan developed a screening assay with the capability of identifying novel antifungal compounds against *A. fumigatus* (64). The authors utilized the release of adenylate kinase (AK) from *A. fumigatus* cell lysis as a proxy to assess the antimold activity of various compounds. AK is a ubiquitously expressed fungal cytosolic enzyme that is released from the cell when it loses its integrity. AK phosphorylates ADP to allow luciferase to drive the formation of light; hence, by adding just two components (ADP and luciferase) to the medium, it was possible to determine the amount of cell lysis by quantifying light production. This AK-luciferase mechanism had originally been established for *S. cerevisiae* as a reporter of autolysis during fermentation (65) but had been used in the pathogenic yeasts *C. albicans* and *C. neoformans* as the basis for high-throughput screens (21, 32). Optimizing this assay for use in *A. fumigatus* allowed the authors to perform a repurposing screen with FDA-approved compounds, validating that this screening assay can identify known antifungal compounds. Moreover, in *A. fumigatus*, this assay allowed researchers to concurrently screen for molecules that prevent conidial germination or cause hyphal lysis. Thus, the dual readout capability of this method makes it an innovative and unique way to study and identify novel anti-*Aspergillus* compounds. This protocol robustly performs in a high-throughput setting and was used to screen several drug libraries, all of which have revealed novel compounds with anti-*Aspergillus* activity. One interesting hit was the molecule PIK-75, found from screening a library of protein kinase inhibitors. This molecule disrupts the integrity of the cell wall in *A. fumigatus* and in *C. neoformans*, but not in *C. albicans* or *S. cerevisiae*, demonstrating that the assay can identify molecules with activity that spans various fungal species. In fact, another hit found in this screen, KAP, was subsequently tested for structure-activity relationships, and several analogs with activity against yeasts, molds, and even some bacteria were found (66). This protocol has many advantages over growth assays traditionally used to screen for compounds with antifungal activity. First, this assay is significantly more sensitive than growth assays which is essential when trying to identify compounds with intermediate activity or when trying to identify the activity of compounds present in extremely small quantities, as would be the case for compounds residing in complex mixtures such as natural product extracts. Secondly, this assay identifies fungicidal compounds, as the AK protocol specifically detects fungal cell lysis. In addition, this technique can be used to identify antifungal compounds with a variety of mechanisms of action, in a more sensitive and targeted way than standard assays used to assess growth. This optimized AK assay is also significant to the field of fungal research since the adaptation of this approach to other molds, such as *Mucor*, *Rhizopus*, *Fusarium*, and *Scedosporium*, is possible. This approach not only revolutionizes the field of antifungal research but also has many implications in fungal genetics since it can allow for the identification of novel mechanisms of action, leading to a better understanding of genetic targets and overall fungal cell biology.

Another way high-throughput assays have been used in model yeast is for morphological profiling (67), looking for molecules that affect the morphology of the fungus under specific genetic or environmental conditions. With recent advances in artificial intelligence and automated recognition, image-based analyses have become more powerful and are a useful strategy to analyze images easily, accurately, and faster than ever. In 2023, Metzner et al. used the freely available CellProfiler software (68) to develop an innovative high-throughput image analysis protocol to distinguish, with high reliability and sensitivity, between filamentous growth and yeast cells at a single-cell level in *C. albicans* (34). In this fungus, the transition from yeast to filamentous growth is known to be one of its major virulence factors (69). Filamentous growth is linked to the secretion of the candidalysin toxin, tissue invasion, induction of macrophage pyroptosis, and biofilm formation, all of which are important for the development of invasive infection (70, 71). The authors then used the pipeline to screen an FDA drug repurposing library and were able to identify over 30 compounds that inhibit yeast filamentation, effectively blocking the yeast-to-hyphal transition. Thus, this innovative approach allowed for the identification of multiple compounds with various mechanisms of action, such as PI3K and TOR inhibitors, known antifungals, and steroids, ultimately identifying phenyl sulfones as novel filamentation inhibitors. Next, using a combination of classical yeast genetic methods to identify resistant mutants, the authors determined the mechanism of action of one of the phenyl sulfone compounds. This compound, called NSC 697923, seems to work by targeting the eIF3 translation initiation complex to inhibit filamentation. Although the translational potential of NSC 697923 is low, since it is toxic to mammalian cells, this study highlights the power of high-throughput automated analysis of microscopy data. Identification of this compound and the successive genetic research following to identify its mechanism of action could potentially be fruitful in the development of other antifungal treatments since it was revealed that the members of the eIF3 complex, which are essential in *C. albicans*, are not conserved in humans. These types of automated image analysis can also be used to monitor the effects of compounds on fungal growth in coculture or assess combination therapies, to identify compounds that may synergistically interact with available antifungal drugs.

#### Adaptation and development of advanced sequencing technologies for fungal pathogens

The use of single-cell mRNA sequencing (scRNA-seq) has led to numerous discoveries in various mammalian cell biology fields, particularly in neuroscience. However, due to some of the technical challenges mentioned above that are presented by fungal cells, scRNA-seq, and related approaches have been unavailable in the fungal field. Excitingly, in 2021, Dohn et al. adapted yeastDrop-seq, a single-cell transcriptomics platform optimized for *S. cerevisiae*, for use in *C. albicans* (24). In the original yeastDrop-seq, also published in 2021, the authors used the single-cell transcriptomics platform in model yeast to identify how guanine and mycophenolic acid alter global transcript levels (72). Most importantly, this novel genetic platform could elucidate heterogenous single-cell gene expression profiles as well as how these profiles are organized in isogenic populations. However, population heterogeneity is expected to be even more pronounced in a commensal organism like *C. albicans*, since it occupies different niches in the human body that are vastly different from each other (such as the mouth, the gut, or the vagina). Hence, applying scRNA-seq to this fungus isolated from their *in vivo* niches could have major implications. As mentioned above, *C. albicans* can undergo a morphological switch from the usual yeast commensal form to the pathogenic filamentous form. Using scRNA-seq on *C. albicans* can help researchers better understand the mechanisms responsible for the commensal-pathogenic switch necessary for causing disease. Moreover, many of the hospital-acquired *Candida* infections are due to biofilm formation on medical devices such as catheters. These biofilms are heterogeneous structures, formed by a three-dimensional structure of yeast, pseudo-hyphae, and hyphae. So, by definition, cells on a single biofilm will be expressing different genes that might be influenced by the environment closest to them. As a result, the authors developed microbial Drop-seq, or mDrop-seq, as a method to perform scRNA-seq in *C. albicans* and other yeast species. To overcome the challenges of yeast cell lysis for genetic research, the authors used thermal incubation and a combination of sarkosyl and zymolyase activities in emulsion drops to optimize this scRNA-seq platform for the yeast species *C. albicans* and *S. cerevisiae*. In addition, they modified the previously developed Drop-seq system to accomplish in-droplet lysis, single-cell microfluidic compartmentalization, and cellular barcoding for the two species of fungi. The authors established the single-cell specificity of mDrop-seq using species-mixing experiments, further validating the use of their approach to understanding yeast genetics. Using this platform, at a single-cell resolution, the authors were able to quantify the transcriptional heterogeneity in different fungal species by performing analysis on a total of 12,012 *S*. *cerevisiae* single cells and 10,314 single cells of *C. albicans*, constructing cluster maps for both species. They were also able to profile the yeasts’ response to environmental stresses, such as fluconazole exposure and heat shock, at single-cell resolution. They found that *C. albicans* cells exposed to the same fluconazole concentration differentially upregulate ribosome activity, iron metabolism, stress response genes like those in ergosterol biosynthesis, and nucleolar and histone activities. They also found that under both stress conditions tested, both yeasts exhibited cell cycle alterations, with fluconazole increasing the number of S-phase cells. Thus, this assay allowed them to uncover novel findings regarding the genetic relationship between cell cycle state and environmental stress. Since mDrop-seq can concurrently profile a mixture of *C. albicans* and *S. cerevisiae* cells, this demonstrates that different fungal species, such as *C. auris*, can be used by this platform, increasing the number of fungal species amenable to single-cell transcriptomic analysis. Moreover, researchers can now elucidate the heterogeneity in how single fungal cells respond to changes in the shifting microbiome or immune system of the host, and if there are, for example, divisions of labor between cells in a population (73). Thus, the development of this technology is a major advance in the field of fungal genetics, as this gives researchers the ability to understand individual yeast transcriptomes in a population for the first time and to analyze the various changes in gene expression that can occur over time in a population in response to a variety of conditions.

Related to advances in sequencing of nucleic acids, high-accuracy gapless assemblies of fungal pathogens’ genomes have been difficult until recently. However, for some medically important fungi, such as *C. tropicalis*, one such genome was absent. While *C. albicans* remains the primary etiological agent of candidiasis, *C. tropicalis* incidence is increasing in tropical countries such as Brazil, India, and Pakistan, where it has become the primary cause of systemic candidiasis acquired in the ICU (74–76). The *C. tropicalis* mortality rate varies from 29% to 72%; however, this is expected to increase in the coming years as the occurrence of multidrug resistance in this pathogen is on the rise (77). In 2020, Guin et al. constructed a genome assembly of *C. tropicalis* at the chromosome level, improving upon the previously available fragmented genome assembly (78). To do this, they employed chromoblots, genetic analysis of aneuploid strains, and third-generation sequencing technologies such as single molecule, real-time sequencing (SMRT-seq) and chromosome conformation capture sequencing (3C-seq). Using 3C-seq data, the authors analyzed the 3D genome organization of *C. tropicalis*, revealing spatial proximity among the telomeres and centromeres of seven chromosomes, uncovering novel insights into the genomic structure of this fungal pathogen. These significant telomere-telomere and centromere-centromere spatial interactions could result in centromeric rearrangements or swapping of chromosomal arms between two interacting chromosomes, which can cause reproductive isolation and promote incipient speciation, highlighting the relevancy of not only understanding the genome sequence but also its spatial organization. In fact, the authors used this data to provide a mechanism by which centromeres evolve but conserve their function. This genome-wide chromatin assembly, named Assembly2020, was the first gapless genome assembly of this fungal pathogen at the chromosome level. Not only that it identified large-scale duplication events and small-scale copy number variations loci within this organism’s genome but that it also mapped indels and SNPs as well as improved annotations of genes and genomic variants that were previously absent in the publicly available fragmented *C. tropicalis* genome assembly, which is overall an invaluable resource for the community. This significant amount of robust data should greatly facilitate *C. tropicalis* genome-wide association studies to provide novel insights into the pathobiology of this fungal pathogen, including the cause of the rising antifungal resistance seen in this species. In broadest terms, the ability to apply SMRT-seq and 3C-seq to fungal pathogens, which allows the sequencing of extremely long reads, will pave the way to study genetic topics that have been traditionally challenging, like centromeres and telomeres, and the spatial organization of the genome, highlighting how advances in genetics can also impact other areas of fungal biology.

#### Adaptation of transformation tools and mutagenesis approaches in emerging pathogens

Highlighting the dormant potential of the fungal kingdom to cause severe disease, in the last years, several new fungal pathogens have emerged or the incidence of known ones has dramatically increased (79). Of them, *C. auris* is of particular importance given its inherent antifungal resistance phenotypes and ability to spread and remain in healthcare settings. However, its study was difficult since there were no forward genetic systems that could be efficiently applied to *C. auris* strains, including a transformation protocol for efficient introduction of DNA. The O’Meara group revolutionized this field in 2021 when they developed tools and approaches for the efficient implementation of both reverse and forward genetics in this fungus (22, 25). The authors were experienced with a method of genome-wide mutagenesis called *Agrobacterium*-mediated insertional mutagenesis (AMIM) that has been applied to a variety of fungal pathogens, including *C. neoformans*, *C. albicans*, and *Histoplasma capsulatum*, and hence decided to adapt and test it on *C. auris*. Their transformation protocol was successful in all four clades of *C. auris*, with the highest efficiency in clade I and the lowest in clade II. Using AMIM in a clade I isolate, the authors performed a screen for altered colony morphology, which is a type of visual screen that has been used countless times in *S. cerevisiae* and *C. albicans*, to identify filamentation regulators. Using this screen, the authors identified six mutants with irregular colony morphology, and five of them showed an aggregating cellular morphology. To validate them, they used their transient, efficient CRISPR system (discussed above) to generate clean deletions of the candidate genes for further study. The authors tested all five genes identified from the AMIM and determined that the irregular colony phenotype was due to defects in cellular separation or changes driving constitutive pseudohyphal growth. Consistent with similar defects in *C. albicans*, when these mutants were tested for virulence, they also exhibited attenuated pathogenesis. Although these phenotypic and molecular discoveries were not surprising, the development of accessible and efficient forward and reverse genetic tools paved the way for the discovery of a unique *C. auris* adhesin that regulates surface colonization and virulence, the two main factors driving the explosive incidence and outbreaks of this fungus (25). Because *C. auris* has been found to persistently colonize the skin of patients and healthcare workers and is known to form biofilms on indwelling medical devices and abiotic surfaces, this ability to attach to surfaces is driving the ongoing outbreaks in healthcare facilities. Studies in other fungi, particularly *C. albicans*, have shown that two families of adhesins, the ALS and IFF/HYR families, mediate these interactions. However, although *C. auris* also encodes members of these families, their role in surface association and colonization was poorly understood. Hence, the O’Meara group used their newly developed tools to discover a previously uncharacterized, and novel, adhesin named surface colonization factor 1 (Scf1). They went on and showed that Scf1, together with a minor role from a conserved adhesin of the IFF/HYR family, is essential for surface colonization, biofilm formation, and virulence. Because these are precisely the traits driving the increased disease burden of *C. auris*, these findings are key in the search of new and improved treatments for this emerging pathogen.

Similarly, *C. glabrata* incidence is increasing and its management is becoming more challenging given the multidrug resistance phenotype exhibited by this species, driving the high mortality rates of infected individuals. Moreover, similar to *S. cerevisiae*, *C. glabrata* strains are important in the biotechnology industry as tools to produce economically important molecules, such as pyruvic acid. However, unlike *S. cerevisiae*, *C. glabrata* lacks a comprehensive molecular toolkit for the genetic manipulation of its genome, which would not only allow for faster studies of the genetic and regulatory pathways governing its transition to virulence but also facilitate the genetic engineering for biotechnological applications. Given that the lack of plasmid vectors has been a technological barrier, many groups have devoted considerable effort to develop them in several fungal pathogens (22, 80), and one example that deserves discussion due to how comprehensive and large it is is the molecular toolkit for *C. glabrata* (CgTK) by Billerbeck and colleagues (63). *C. glabrata* is closer to *S. cerevisiae* in the evolutionary tree than all the other members of the *Candida* CTG clade (named for the changes in the translation of the CTG codon); hence, the authors reasoned that the most efficient way to build a CgTK was to use the existing *S. cerevisiae* toolkit as a starting point. In the end, the authors developed and validated a series of preassembled vectors with four auxotrophic markers or one dominant marker, with various promoters and plasmid-copy numbers to control the level of expression, including three inducible/repressible promoters, three degradation tags, and the CRISPRi system discussed above. We agree with the authors that the creation of this CgTK will drive and accelerate the genetic studies and phenotypic engineering of *C. glabrata* in a way akin to how the genetic toolkit for *S. cerevisiae* led the way in the field in the early 2000’s.

## CONCLUSION

Although it might not be common knowledge for the general audience, fungi have been instrumental in the well-being of humanity for centuries (think on wine, bread, and antibiotics). Moreover, with the development of genetic techniques for *S. cerevisiae*, we were able to produce important drugs and medicines such as insulin and unravel the complexities of how our own cells function, leading to a better understanding of cellular processes and how to correct them when they go awry. With the increasing threat of fungal pathogens, it is critical that we develop similar genetic techniques specific for these “not-so-useful” fungi to understand how they can cause disease and thus identify ways to prevent or treat it. Looking back at our original commentary (1), we believe that we are on track to develop genetic techniques akin to the heyday of *S. cerevisiae*, and these advanced genetic techniques are keeping the field of fungal pathogenesis stronger than ever.
